# Effects of Real-Ambient PM_2.5_ Exposure on Lung Damage Modulated by Nrf2^−/−^

**DOI:** 10.3389/fphar.2021.662664

**Published:** 2021-04-23

**Authors:** Hao Ding, Menghui Jiang, Daochuan Li, Yanjie Zhao, Dianke Yu, Rong Zhang, Wen Chen, Jingbo Pi, Rui Chen, Lianhua Cui, Yuxin Zheng, Jinmei Piao

**Affiliations:** ^1^ School of Public Health, Qingdao University, Qingdao, China; ^2^ Department of Toxicology, School of Public Health, Sun Yat-sen University, Guangzhou, China; ^3^ Department of Toxicology, School of Public Health, Hebei Medical University, Shijiazhuang, China; ^4^ School of Public Health, China Medical University, Shenyang, China; ^5^ School of Public Health, Capital Medical University, Beijing, China

**Keywords:** Nrf2, CYP450, lung, PM2.5, endoplasmic reticulum stress

## Abstract

Previous studies have shown that long-term exposure to fine particulate matter (PM_2.5_) increases the morbidity and mortality of pulmonary diseases such as asthma, chronic obstructive pulmonary disease and pulmonary emphysema. Oxidative stress and inflammation play key roles in pulmonary damage caused by PM_2.5_. Nuclear factor erythroid 2-related factor 2 (Nrf2) could regulate the expression of antioxidant and anti-inflammatory genes and is pivotal for protection against PM_2.5_-induced oxidative stress. In this study, a real-ambient exposure system was constructed with the outdoor ambient air in north China. Wild-type (WT) and Nrf2^−/−^ (KO) mice were exposed to the real-ambient system for six weeks. After PM_2.5_ exposure, our data showed that the levels of inflammatory factors and malondialdehyde were significantly increased in WT and KO mice. Moreover, the lung function and pathological phenotype of the WT mice were altered but there was no obvious change in the Nrf2^−/−^ mice. To further explore the potential molecular mechanisms, we performed RNA-sequencing. The RNA-sequence analysis results showed that the CYP450 pathway in the first ten pathways of KEGG was related to the metabolism of PM_2.5_. In WT and KO mice, the expression of CYP2E1 in the CYP450 pathway showed opposite trends after PM_2.5_ exposure. The data showed that the expression of the CYP2E1 gene in WT-PM mice increased while it decreased in KO-PM; the expression of the CYP2E1 protein showed a similar trend. CYP2E1 is primarily distributed in the endoplasmic reticulum (ER) where it could metabolize various exogenous substances attached to PM_2.5_ and produce highly toxic oxidation products closely related to ER stress. Consistently, the expression level of GRP94, a biomarker of ER stress, was increased in WT mice and reduced in KO mice under PM_2.5_ exposure. Persistent ER stress is a mechanism that causes lung damage under PM_2.5_ exposure. Nrf2 facilitates lung injury during PM_2.5_ exposure and CYP2E1 metabolism is involved in this process.

## Introduction

PM_2.5_ is fine ambient particulate matter with aerodynamics diameter <2.5 μm. Due to its small particle size and large specific surface area, a large number of toxic substances such as heavy metals and microorganisms are adsorbed on its surface; they can reach the deep parts of the human respiratory tract and deposit in the alveoli through respiration (Kirrane et al., 2019). PM_2.5_ exposure can induce or aggravate diseases such as pulmonary inflammation (Sint et al., 2008), asthma, and atherosclerosis (Yang et al., 2019). Accumulating evidence has confirmed that inflammatory responses and oxidative stress are involved in PM_2.5_-induced pulmonary disease, including chronic obstructive pulmonary disease and pulmonary emphysema (Gu et al., 2017; Wang et al., 2019).

Nuclear factor E2-related factor 2 (Nrf2) is an important transcription regulator that belongs to the family of bZIP and is a key antioxidant response gene that could regulate the expression of many antioxidant genes (Buendia et al., 2016; Ahmed et al., 2017). Previous studies have demonstrated in both *in vivo* and vitro experiments that PM_2.5_ could activate the expression of Nrf2, which could then regulate oxidative stress and inflammation (Jin et al., 2019; Sun et al., 2020). Nrf2 has a wide range of biological functions, including the ability to affect the metabolism of CYP450 enzymes (Abu-Bakar et al., 2007; Shen and Kong, 2009; Ashino et al., 2014).

Cytochrome P450 (CYP450) is a general term for a series of heme-thiolate proteins that play an important role in the metabolic process. CYP450 could metabolize the polycyclic aromatic hydrocarbons attached to PM_2.5_ and produce ROS during their metabolism, which can cause oxidative stress and lung damage. As a member of the phase I detoxification enzyme system, CYP2E1 is primarily distributed in the endoplasmic reticulum (ER) and mitochondrial membrane and can metabolize various endogenous and exogenous substances (Son et al., 2017). A recent study showed that the liver damage in CYP2E1-knockout mice was significantly less than in WT mice after exposure to N, N-dimethylformamide (DMF). These results suggest that ER stress is decreased in the KO mice because the toxic metabolites of DMF are reduced by the loss of CYP2E1 (Wu et al., 2019b).

Traditional animal models of PM exposure typically use intratracheal instillation or concentrated ambient particles (CAPs) systems to introduce the pollutants (Su et al., 2017; Lin et al., 2018). Intratracheal instillation will change the physical and chemical properties of particulate matter, and the CAPs exposure system transforms the size of the particles and increases the negative pressure in cages. To represent real-ambient exposure, this study used an individually ventilated cage (IVC) system that almost completely simulates the real-ambient exposure of PM_2.5_ under outdoor atmospheric PM_2.5_ pollution (Li et al., 2019a; Li et al., 2019b; Chu et al., 2019; Li et al., 2020a; Cui et al., 2020). The composition and exposure dose of PM is also accurately recorded. Therefore, this real-ambient exposure system made up for the shortcomings of unclear exposure dose and the concentration of PM_2.5_ in traditional epidemiological studies and could better explain the results and provide valuable clues for disease prevention.

In the present study, we investigated the role of Nrf2 in PM_2.5_-induced lung damage by applying a real-ambient exposure system. These findings provide new evidence about the role of Nrf2 in lung injury caused by PM_2.5_ exposure.

## Materials and Methods

### Animal

Our experiment used Nrf2-knockout and wild-type mice bred from C57BL/6J mice as animal models. The Nrf2^−/−^ mice were developed by Prof. Masayuki Yamamoto of Tohoku University and offered by the lab of Jingbo Pi, China Medical University. We used the female and male Nrf2 heterozygous (Nrf2^+/−^) mice to breed the Nrf2^−/−^ (KO) mice. And the wild-type (WT) mice were littermate Nrf2^+/+^ mice in the experiments. We used PCR to characterize the offspring of mice according to previous protocol (Jiang et al., 2020a). The expression of Nrf2 protein in different groups was showed in the Supplemental Material. The procedures for care and use of animals were approved by the Ethics Committee of the Qingdao University and all applicable institutional and governmental regulations concerning the ethical use of animals were followed.

### Real-Ambient Exposure System

This study used a real-ambient exposure system; the exposure device is located in Shijiazhuang. The PM_2.5_ concentration in this city far exceeds the mean daily recommended dose limit in China. In winter, the main pollution source in this city is burning coal. The installation of exposure was described in a previous study (Li et al., 2019a; Jiang et al., 2020b). In brief, the unique system consists of control and exposure chambers. And the control chambers were connected to high efficiency particulate air (HEPA)-filtered air (FA), while the exposure chambers were ventilated with unfiltered outdoor air (PM). The factors in all chambers were identical, including temperature, humidity, pressure, ventilation frequency, and noise. The WT and KO mice were exposed to FA or PM chambers (sixteen in each chamber), respectively. The mice could access to standard food and water freely in the chambers with a 12 h light/dark cycle. The time of mice exposure to PM_2.5_ or FA was 24 h/day and 7 days/week for 6 weeks. PM concentration in FA and PM chambers was monitored daily using the Aerosol Detector DUSTTRAKTM II and analyzed the size of particle with an Aerodynamic Particle Sizer Spectrometer 3321 (TSI Incorporated, Shoreview, MN, United States). During the 6-week exposure period, there are 12 days when the exposure concentration exceeds 150 μg/m^3^ in the exposure chamber (Li et al., 2019a). Our previous study showed the average daily PM_2.5_ concentration in the air around the study site was 151.40 μg/m^3^, while the average PM concentration in the exposure chamber was 89.95 μg/m^3^ (Jiang et al., 2020a). According to our previous method, the cumulative amount of PM_2.5_ inhaled into pulmonary was calculated. The cumulative amount of PM_2.5_ inhaled into pulmonary was 87.04 μg after exposed for 6 weeks (Jiang et al., 2020b).

### Histopathological Analysis

The lung tissues were extracted from the mice and washed with phosphate buffered saline (PBS). After fixed in 4% formaldehyde for 24 h at the room temperature, dewatered by graded ethanol, implanted in paraffin, and sectioned transversely. Tissue sections were deparaffinized and stained with hematoxylin and eosin (H&E). The histological assessment was achieved under the light microscope. The histopathological analysis of acute lung injury were quantified using a scoring system described in a previous study (Matute-Bello et al., 2011). Five regions were randomly selected from each pathological section, and the inflammatory cells in each region were quantified with ImageJ (NIH, United States) software.

### RNA-Seq Assay

Three lung tissue samples were randomly selected from per group (WT-FA, WT-PM, KO-FA, and KO-PM) and carried on RNA-sequencing test. The assay was performed by BioMiao Biological Technology (Beijing, China). A total amount of 3 μg RNA per sample was used as input material for the RNA sample preparations. Sequencing libraries were generated using NEBNext®Ultra™ RNA Library Prep Kit for Illumina® (NEB, United States) following manufacturer’s recommendations and index codes were added to attribute sequences to each sample. Briefly, mRNA was purified from total RNA using poly-T oligo-attached magnetic beads. Fragmentation was carried out using divalent cations under elevated temperature in NEBNext First Strand Synthesis Reaction Buffer (5X). First strand cDNA was synthesized using random hexamer primer and M-MuLV Reverse Transcriptase (RNaseH-). Second strand cDNA synthesis was subsequently performed using DNA Polymerase I and RNase H. Remaining overhangs were converted into blunt ends via exonuclease/polymerase activities. After adenylation of 3′ ends of DNA fragments, NEBNext Adaptor with hairpin loop structurewere ligated to prepare for hybridization. To select cDNA fragments of preferentially 150–200 bp in length, the library fragments were purified with AMPure XP system (Beckman Coulter, Beverly, United States). Then 3 μl USER Enzyme (NEB, United States) was used with size-selected, adaptor-ligated cDNA at 37°C for 15 min followed by 5 min at 95 C before PCR. Then PCR was performed with Phusion High-Fidelity DNA polymerase, Universal PCR primers andIndex (X) Primer. At last, PCR products were purified (AMPure XP system) and library quality was assessed on the Agilent Bioanalyzer 2100 system. The clustering of the index-coded samples was performed on a cBot Cluster Generation System using TruSeq SR Cluster Kit v3-cBot-HS (Illumia) according to the manufacturer’s instructions. After cluster generation, the library preparations were sequenced on an Illumina Hiseq 2000/2500 platform and 150 bp/100 bp/50 bp paired/single-end reads were generated. A differential expression analysis of two groups was achieved by the DESeq2 R package. KEGG enrichment pathways over the differential expression genes were performed by the Cluster Profiler R package.

### RT-PCR

Total mRNA was extracted from 50 mg lung tissues of mice with the Trizol agent (Thermo Scientific, Waltham, United States) according to the instructions of manufacturer-provided. cDNA was synthesized using the reverse transcription kit (Takara, Kyoto, Japan). And performed quantitative real time PCR (qRT-PCR) using a SYBR Green PCR Master Mix (Thermo Fisher Scientific, Waltham, United States) with QuantStudio seven Real-Time PCR Systems (Thermo Scientific, Waltham, United States). The level of each gene expression was adjusted to the β-actin. The method of calculate relative expression of genes was 2^–ΔΔCt^. The primers used are described in the Supplemental Material.

### Western Blotting

The lung samples of mice were homogenized in a RIPA buffer (Solarbio, Beijing, China) with phenylmethylsulfonyl fluoride (PMSF) and alkaline phosphatase inhibitor cocktail (Solarbio, Beijing, China). The protein concentration was determined using the Bicinchonic acid (BCA) protein analysis kit (Epizyme, Shanghai, China) according to its protocol. Then, 40–60 μg protein from lungs were separated using 10% SDS-Polyacrylamide-Gel-Electrophoresis (SDS-PAGE) followed by transfer to polyvinylidene fluoride (PVDF) membranes (Millipore, Billerica, MA, United States). The PVDF membranes were blocked with defatted milk for 3 h at room temperature, then incubated with specific primary antibodies including CYP2E1 (Affinit, Beijing, 1:1,000), Nrf2 (Cell Signaling Technology, Boston, 1:1,000), GRP94 (Affinit, Beijing, 1:1,000), CHOP (Affinit, Beijing, 1:1,000), GAPDH (Bioss, Beijing, 1:3,000) overnight at 4°C. After washing four times with TBST (15 min per time), the goat anti-rabbit IgG secondary antibody (Epizyme, Shanghai, China) were incubated for 1 h at room temperature. Followed by additional three times washing, the membranes were detected using automatic chemiluminescence image analysis system (Millipore, Billerica, MA, United States) and quantified with ImageJ (NIH, United States) software.

### Immunohistochemistry and TUNEL Assay

The expression levels of GRP94 and CHOP were evaluated with immunohistochemical staining of the aortic tissue. Sections of lung tissue were incubated with GRP94 (Affinit, Beijing, 1:1,000) or CHOP (Affinit, Beijing, 1:1,000) antibodies at 37°C for 1 h, washed with PBS (pH 7.4) and then incubated with secondary antibody for 20 min. Sections were treated with Biotin-labeled Goat Anti-Rabbit IgG, developed with freshly prepared DAB solution, and counterstained with hematoxylin. Pictures were taken with a microscope (Changfang, Shanghai, China), and quantified using a scoring system described in a previous study (Li et al., 2019b). Terminal deoxynucleotidyl transferase dUTP nick end labeling (TUNEL) assay kit was used to detect apoptotic cells in lung tissue sections following manufacturers’ instructions. Sections were counter stained with DAPI. Five regions were randomly selected from each section, and the TUNEL positive cells in each region were quantified with ImageJ (NIH, United States) software.

### Malondialdehyde and Superoxide Dismutase Assay

The MDA and SOD concentration of lung tissue samples was determined using a special kit (Solarbio, Beijing, China). Briefly, 100 mg homogenate of lung tissues were mixed with 200 μl MDA detection solution and 600 μl MDA working solution. Subsequently, the mixture was incubated at 100°C for 1 h according to the instructions of the kit. The mixture was centrifuged at 15,000 r for 15 min using centrifuge. Then, 200°µl of the supernatant was moved to a 96-well plate to read absorbance at 450, 532, and 600 nm using a microplate reader. And the concentration of MDA in lung was calculated by the absorbance according to the instructions of kit. SOD assays were performed using assay kits, according to manufacturers’ instructions.

### Statistical Analysis

Statistical analysis was performed with GraphPad Prism (8.0) software. Data were presented as mean ± SEM. A factorial design two-way analysis of variance (TWO WAY-ANOVA) was used to assess differences among groups. Results were considered statistically significant when *p* < 0.05.

## Result

### Effects of Nuclear Factor Erythroid 2-Related Factor 2 on Pulmonary Function and Pathology In PM_2.5_-Exposed Mice

**FIGURE 1:**
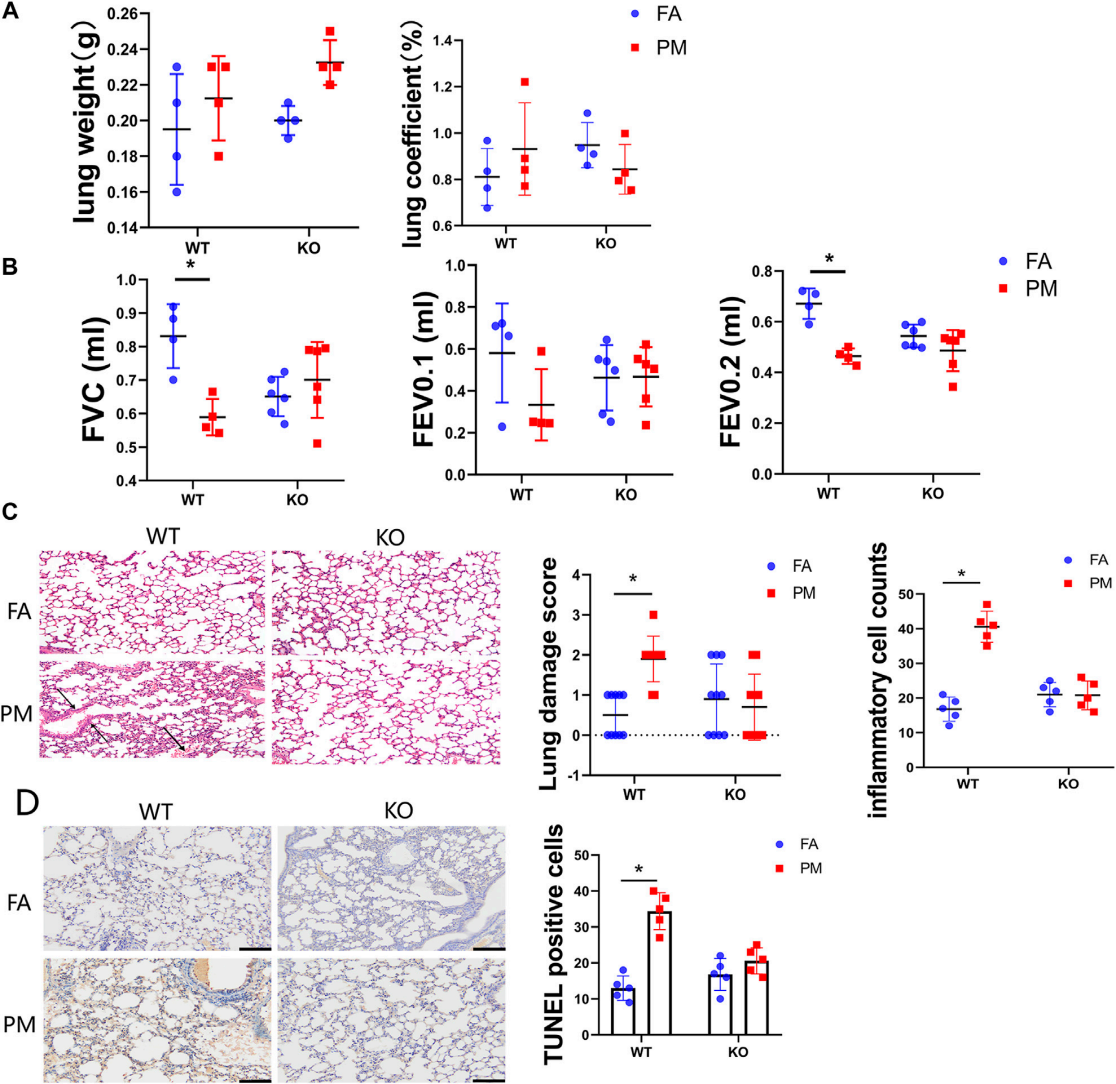
Effects of Nrf2 knockout on pulmonary function and pathology in PM_2.5_-exposed mice. **(A)** The lung weight and ratio of lung to body weight of four groups mice after the treatment. *n* = 4 per each group. **(B)** The tracheas of mice were connected to the instrument measuring pulmonary function under anaesthesia (*n* = 6), and the indicators of lung function were monitored by instrument. Lung function parameters: FVC, forced vital capacity; FEV0.1, forced expiratory volume in the first 0.1°s; FEV0.2, forced expiratory volume in the first 0.2°s. **(C)** Lung was isolated, fixed, sectioned, and observed microscopically after hematoxylin and eosin (H&E) staining (representative of four mice, magnification ×200). The lung injury scores and inflammatory cells counts were calculated in different groups of mice. **(D)** Lung tissue sections from four groups mice were isolated and test the apoptosis cells by TUNEL assay kit. The TUNEL positive cells were quantified with ImageJ (NIH, United States) software. FA, filtered air; PM, fine particulate matter. WT, wild type mice; KO, Nrf2^−/−^ mice. Scale bars are 50°μm, and magnification is ×200. Data are expressed as the mean ± SEM, *p < 0.05.

To study the effects of PM_2.5_ on the lung, mice were exposed to the real-ambient system. During PM exposure, no significant changes were observed in the body weight of the four groups mice (Jiang et al., 2020a). After PM_2.5_ exposure, no remarkable difference was observed in the lung weight or the ratio of the lung-to-body weight between the WT-FA and WT-PM groups or the KO-FA and KO-PM groups (Figures 1A,B).

To assess the effects of PM exposure on the lung, we conducted lung function tests on the mice. The forced vital capacity (FVC) and forced expiratory volume in the first 0.2 s (FEV0.2) were significantly decreased in the WT-PM group relative to the WT-FA group, while no remarkable difference was detected between the KO-FA and KO-PM groups (Figure 1B). However, the forced expiratory volume in the first 0.1 s (FEV0.1) displayed no statistically significant between groups difference (Figure 1B). Taken together, these results suggest that PM_2.5_ leading to pulmonary injure in WT mice and Nrf2 loss ameliorated lung damage under PM_2.5_ exposure. To appraise the effects of PM_2.5_-induced lung injury, we conducted histopathological assessments. In wild-type mice, the pathological tests suggested serious alveolar congestion and alveolar wall thickening after PM exposure. The typical manifestations of lung damage were alveolar congestion and alveolar wall thickening. Interestingly, there was no significant pathological change between the KO-FA and KO-PM groups (Figure 1C). To better characterize the degree of lung injury in the course of PM exposure, we quantified lung injury based on the histopathological features as described previously (Matute-Bello et al., 2011). And the inflammatory cell counts data in different groups were presented. To evaluate whether PM2.5 could induce apoptosis in the lung tissue of WT and KO mice, we decided to observe the apoptotic cells using TUNEL assay (Figure 1D). Consistent with pathological test results, PM_2.5_ exposure significantly increased apoptotic cells in WT mice; however, there was no difference between the KO-FA and KO-PM mice. Collectively, the results suggested that PM_2.5_ exposure led to lung damage of WT mice rather than KO mice.

### PM_2.5_ Exposure Enhanced the Inflammation and Oxidative Stress of the Lung in the Wild-Type and KO Mice

**FIGURE 2:**
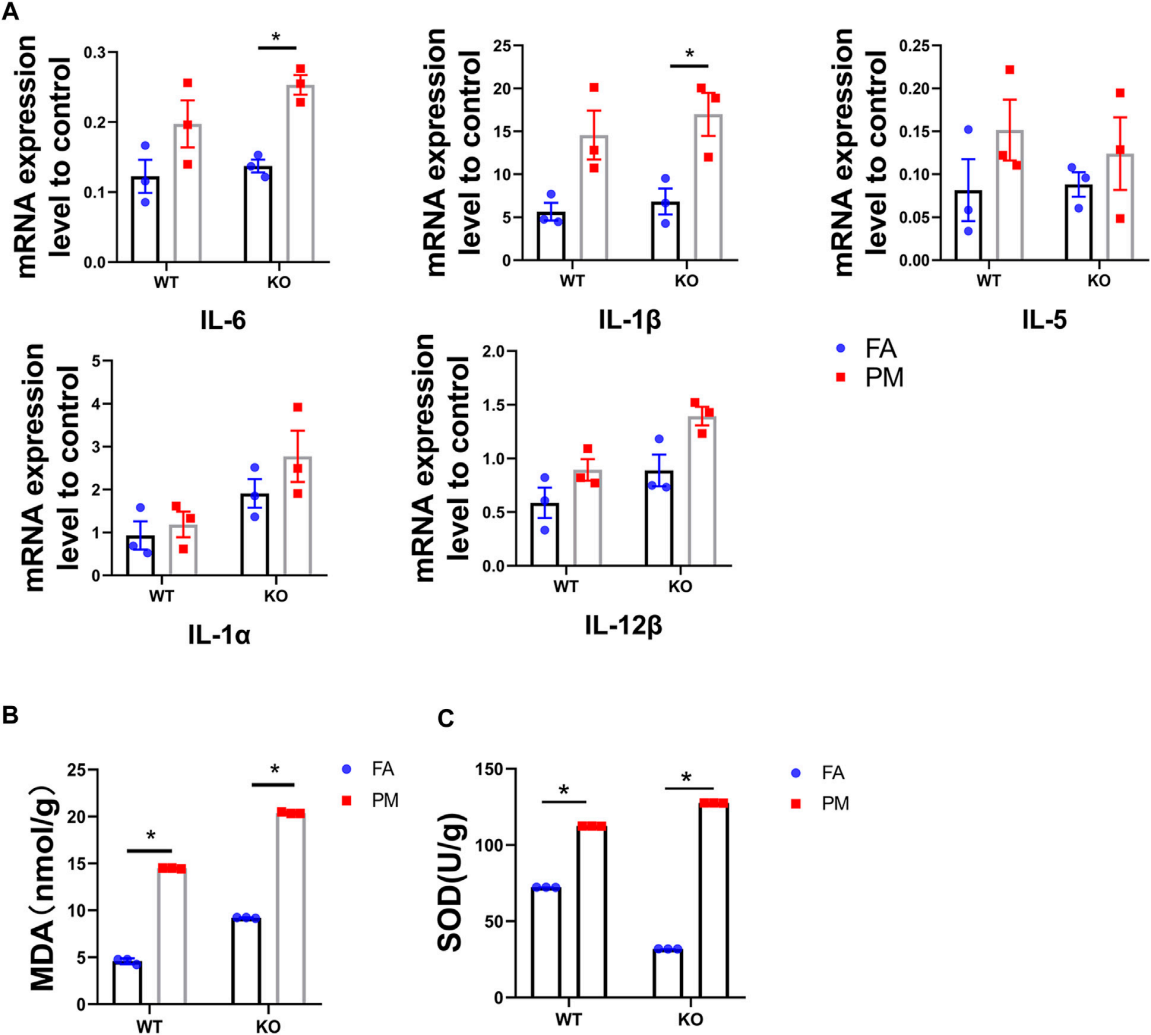
Nrf2 knockout exacerbates PM_2.5_-induced inflammation and oxidative stress in lung. **(A)** The expression level of inflammatory factors in four groups (*n* = 3). **(B)** MDA contents in four groups. *n* = 3 per group. **(C)** SOD contents in four groups. *n* = 3 per group. FA, filtered air; PM, fine particulate matter. WT, wild type mice; KO, Nrf2^−/−^ mice. Data are expressed as the mean ± SEM, *p < 0.05.

The Keap1-Nrf2 signaling pathway plays a vital role in protecting cells from inflammation and oxidative stress (Ruiz et al., 2013). In hence, we determined the inflammation and oxidative stress levels in the lungs of WT and KO mice after exposure to PM_2.5_ for 6 weeks. The mRNA levels of IL-6 and IL-1β in the WT and KO mice were all obviously increased after PM exposure compared to the FA group (Figure 2A). Although the changes in the mRNA expression levels of IL-1α and IL-5 were not statistically different, they showed the same trend as IL-6 and IL-1β. Subsequently, we assessed the effects of Nrf2 knockout on the oxidative stress response upon PM exposure. Treatment with PM_2.5_ for six weeks increased the levels of MDA and SOD in the lungs of WT and KO mice (Figures 2B,C). Nrf2 can regulate the expression of many antioxidant genes, including HO-1, NQO1 and GCLC. Our study tested the expression of HO-1, NQO1 and GCLC (Supplemental Material), and there was a significant down-regulation in the Nrf2 knockout mice, while the PM_2.5_ exposure seemed to effectively increase the expression levels in WT-PM mice. These results showed that PM_2.5_ exposure could promote inflammation and oxidative stress in the lung in both WT and KO mice.

### The CYP450 Pathway is Involved in Lung Damage in KO Mice After Exposure to PM_2.5_

**FIGURE 3:**
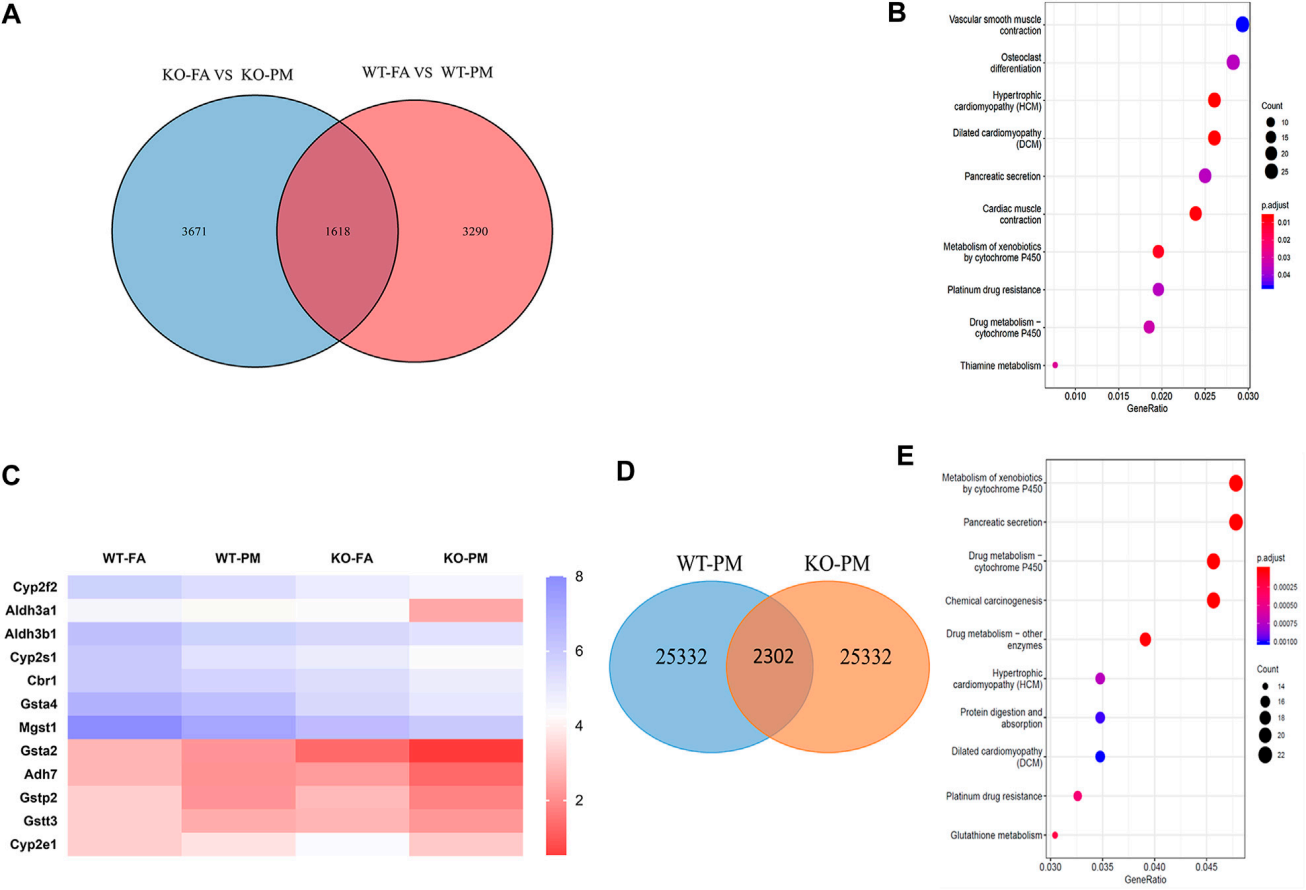
CYP450 pathway in involved in lung damage of KO mice after exposure to PM_2.5_. **(A)** Venn diagram showing the number of genes that fold change >2 shared by WT-FA *vs.* WT-PM and KO-FA *vs.* KO-PM. *n* = 3 per group **(B)** KEGG enrichment of 3,671 genes in four groups mice. *n* = 3 per group **(C)** Heatmap for all the samples of “Metabolism of xenobiotics by cytochrome P450” pathway. *n* = 3 per group **(D)** The Venn diagram of 2,302 DEGs were identified in KO-PM mice relative to WT-PM mice. *n* = 3 per group **(E)** The top-10 KEGG pathway enrichment of 2,302 DEGs were identified in KO-PM mice relative to WT-PM mice. *n* = 3 per group. FA, filtered air; PM, fine particulate matter. WT, wild type mice; KO, Nrf2^−/−^ mice.

To further explore the mechanism of lung damage induced by Nrf2 knockout after exposure to PM_2.5_, we used the lung tissue of the mice for RNA-seq analysis. The RNA-seq results suggested that thousands of genes had been changed by Nrf2 knockout or PM exposure. The selection criteria of differentially expressed genes (DEGs) were fold-change greater than twofold and *p*-value <0.05 in different groups. The numbers of DEGs among the different groups are shown in Figure 3A. The initial analysis screened out 4,908 and 5,289 DEGs based on the comparison between WT-FA and WT-PM and between KO-FA and KO-PM. Of these, 1,618 genes were confirmed as shared by the two parts. To determine the influence of Nrf2 loss, we removed 1,618 of the 5,289 genes and studied the remaining 3,671 genes. These 3,671 genes were subjected to top-10 KEGG pathway enrichment analysis (Figure 3B). Major KEGG signaling pathways observed with significant changes included vascular smooth muscle contraction, osteoclast differentiation, hypertrophic cardiomyopathy, dilated cardiomyopathy pathways, and metabolism of xenobiotics by cytochrome P450 (CYP450). In those pathways, major pathways were associated with cardiovascular disease, osteoclast differentiation and CYP450 enzyme; Plenty studies showed that the expression of CYP450 was related to the metabolism of various exogenous compounds has an impact on the lung damage caused by PM_2.5_ (Saint-Georges et al., 2008; Abbas et al., 2009; Oesch et al., 2019), we chose to further explore the “Metabolism of xenobiotics by cytochrome P450” pathway. This pathway enrichment contains 18 genes and their *p*-value was 0.01. The heatmaps of all samples in this pathway are shown in Figure 3C. Meanwhile, 2,302 DEGs were identified in KO-PM mice relative to WT-PM mice and the 2,302 genes were subjected to top-10 KEGG pathway enrichment analysis (Figures 3D,E). The top 10 KEGG pathways are mainly related to metabolism, including metabolism of xenobiotics by cytochrome P450. The metabolism of xenobiotics by the cytochrome P450 signaling pathway mainly involves the genes encoding the phases I (CYP2E1, CYP2F2, CYP2S2, ALDH3A1, ALDH3B1, ADH7, and CBR1) and II (GSTA2, GSTA4, GSTP2, GSTT3, and MGST1) metabolic enzymes among these 3,671 genes. The expression of phase I metabolic enzyme-related genes was down-regulated in the WT and KO mice following exposure to PM_2.5_. Phase I metabolic enzymes could metabolize organic components in PM_2.5_ such as polycyclic aromatic hydrocarbons (PAHs) and release more toxic oxidation products that impact lung function.

### Expression of CYP450 Pathway-Related Genes in the Four Groups of Mice

**FIGURE 4:**
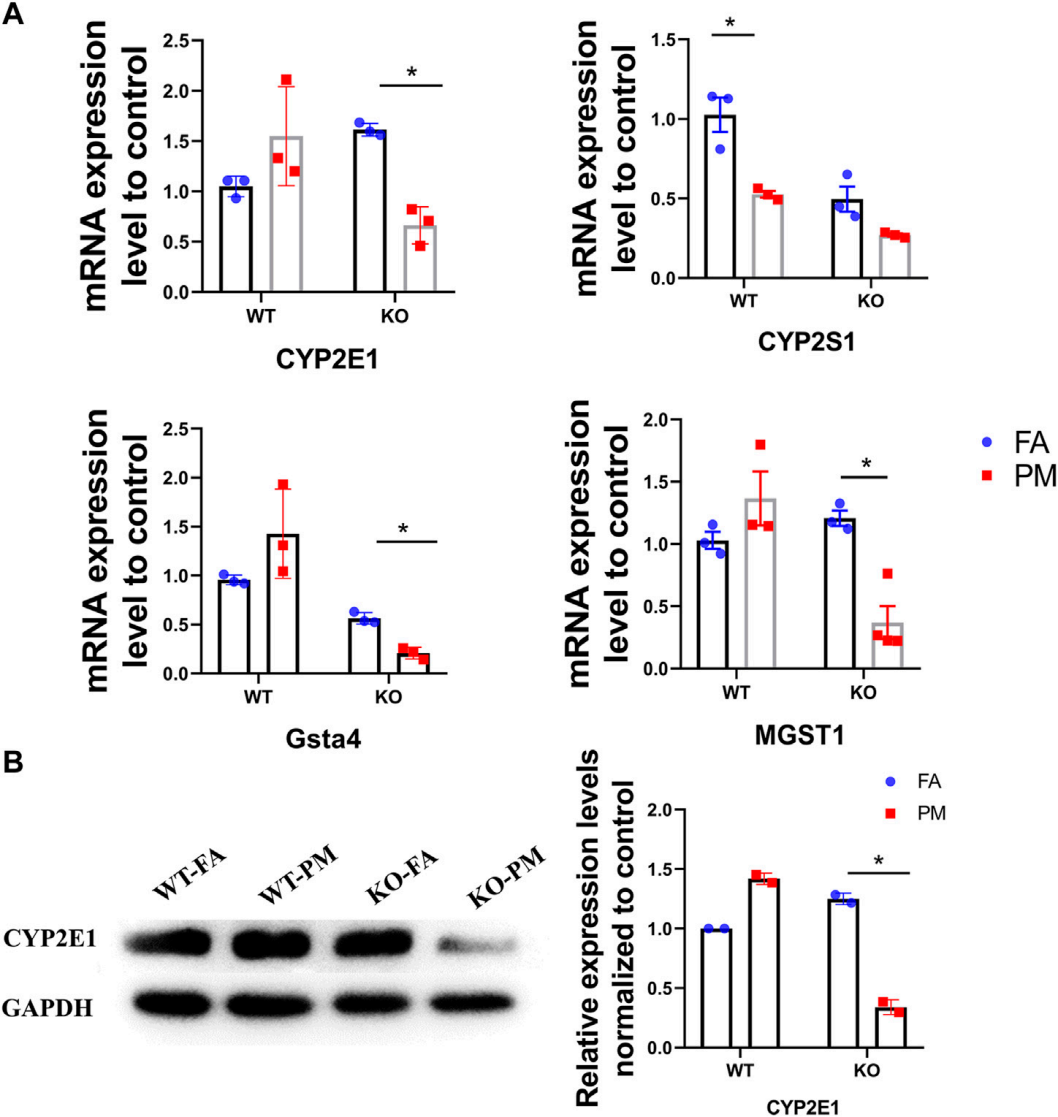
Expression of CYP450 pathway related genes in four groups of mice. **(A)** The expression of main genes in the “Metabolism of xenobiotics by cytochrome P450” pathway. *n* = 3 per group. **(B)** The CYP2E1 protein expression levels in four groups of mice were analyzed by Western blot and the quantification of the CYP2E1 expression. *n* = 2 per group.

CYP450 expression can be generally regulated by many factors including xenobiotics such as acetaminophen, PAHs, and drugs (Guengerich et al., 2016; Yao et al., 2019) and it could metabolize the polycyclic aromatic hydrocarbons attached to PM_2.5_ and produce ROS during this metabolism, which can cause oxidative stress and lung damage. Under exposure to PM_2.5_, the mRNA expression levels of CYP2E1, HPGDS, and UGT1A7C in the WT mice were increased. However, the mRNA expression levels of CYP2E1, CYP2S1, GSTA4, and MGST1 in the Nrf2-knockout mice were down-regulated after PM_2.5_ exposure (Figure 4A). Moreover, we observed a remarkable decrease in the protein levels of CYP2E1 expression in the PM_2.5_-treated lungs of Nrf2-knockout mice, but not in WT mice (Figure 4B). CYP450 enzymes participate in the phase I metabolism of chemical toxins and poisons in the body and could produce highly toxic oxidation products (Saint-Georges et al., 2008). Our results showed that the CYP2E1 was decreased in KO mice after exposure to PM_2.5_. Based on this result, we speculate fewer toxic products were produced in the KO-PM mice, which led to less damage to the lung. In addition, the decreased expression of CYP2E1 may lead to lower ER stress levels (Torres et al., 2019).

### Expression Level of Glucose-Regulated Protein 94 and C/EBP Homologous Protein, Biomarkers of Endoplasmic Reticulum Stress

The CYP450 enzyme is rich in the ER, which is an important organelle involved in detoxification, protein synthesis, modification, and folding (Brignac-Huber et al., 2016). The metabolic activation of toxins by CYP2E1 can cause an imbalance in the homeostasis of the ER, leading to unfolded and misfolded proteins aggregating in the reticulum cavity to induce ER stress (Lewis and Roberts, 2005). ER stress participates in the toxic effects of various environmental and occupational toxicants on cells (Wang and Tang, 2020). When the ER stress response is too forceful or the stimulation time too long, it will induce cell apoptosis and cause body damage (Fernández et al., 2015; Xie et al., 2019). ER stress was shown to be activated in PM_2.5_-induced lung and liver injury and is an important mechanism by which PM_2.5_ causes lung damage (Laing et al., 2010).

**FIGURE 5:**
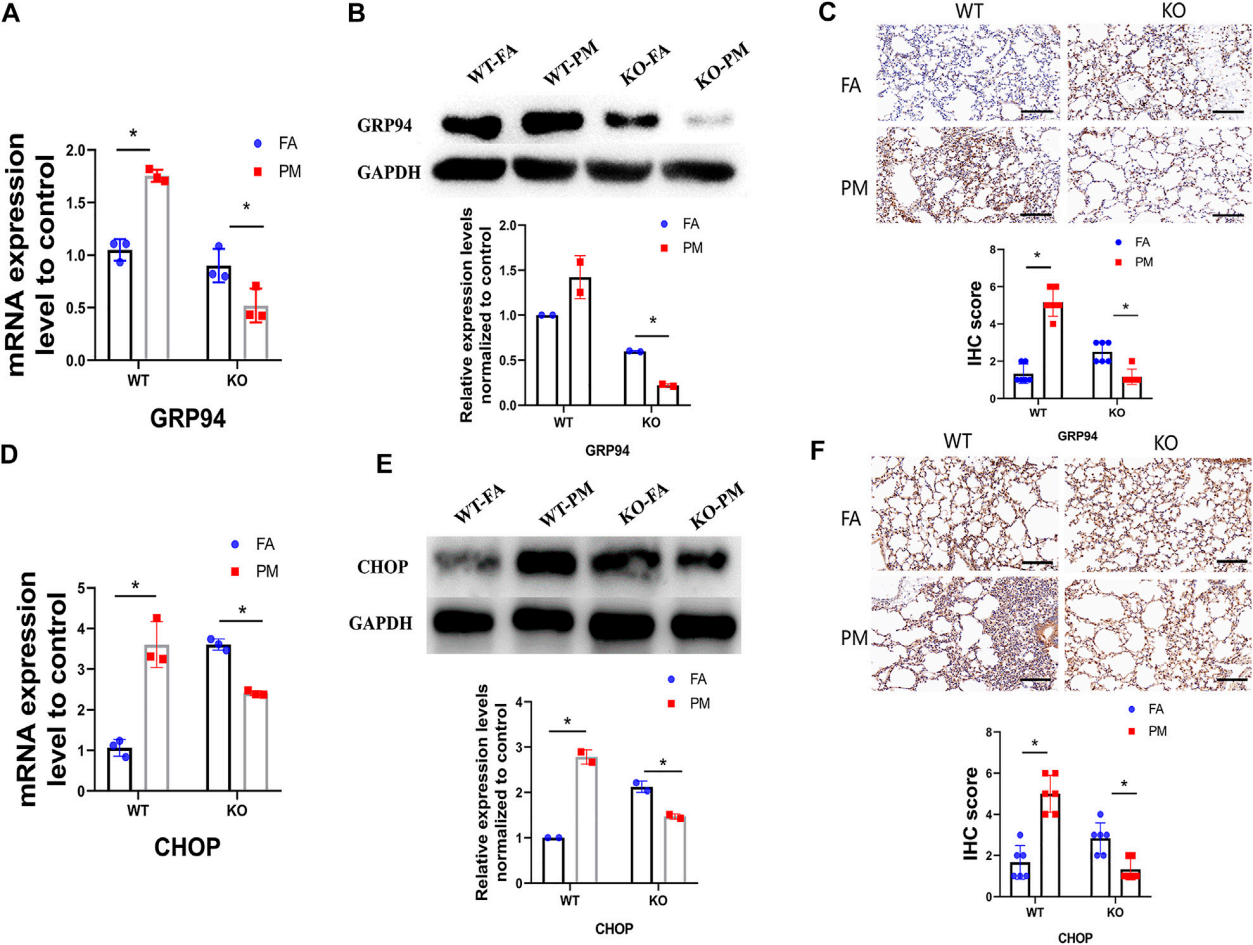
The expression level of GRP94 and CHOP, biomarkers of ER stress. **(A)** Total RNA was extracted from lung tissues, and the mRNA levels of GRP94 was detected by QPCR. *n* = 3 per group. **(B)** The GRP94 protein expression levels in four groups of mice were analyzed by Western blot and the quantification of the GRP94 expression. *n* = 2 per group. **(C)** After exposure, IHC staining of GRP94 was performed in lung tissues, and the IHC score of GRP94 expression was calculated. *n* = 3 per group. **(D)** The mRNA level of CHOP was detected by QPCR. *n* = 3 per group. **(E)** The CHOP protein expression levels in four groups of mice were analyzed by Western blot and the quantification of the CHOP expression. *n* = 2 per group. **(F)** The IHC staining of CHOP was performed in lung tissues and the IHC score of CHOP expression was calculated. *n* = 3 per group. FA, filtered air; PM, fine particulate matter. WT, wild type mice; KO, Nrf2^−/−^ mice. Scale bars are 50 μm, and magnification is ×300. Data are expressed as the mean ± SEM, *p < 0.05.

GRP94 and CHOP play pivotal roles in maintaining protein homeostasis, participating in the ER unfolded protein response, and is involved in ER-related protein degradation. Therefore, GRP94 and CHOP were used as biomarkers of ER stress. To explore whether ER stress contributes to PM_2.5_-induced lung toxicity, GRP94 and CHOP was measured in the lungs of the four different groups of mice (Figures 5A–F). Compared with the WT-FA group mice, the expression levels of GRP94 and CHOP were high in the wild-type mice after exposure to PM_2.5_, while a significant decrease of GRP94 and CHOP expression was observed in the KO-PM mice compared to the KO-FA mice, suggesting that Nrf2 knockout leads to ER stress reduction following exposure to PM_2.5_.

## Discussion

Shijiazhuang, located in the north of China, is one of five cities with the highest PM_2.5_ concentration in China. The air pollution there mainly comes from the burning of coal, industrial emissions, and vehicle exhaust (Yang et al., 2013). Therefore, we built a real-ambient exposure system in Shijiazhuang city. Traditional animal models of PM exposure typically use intratracheal instillation, which changes both the physical and chemical properties of particulate matter. To represent real-ambient exposure, our study used a real-ambient exposure system that almost completely simulates the real-ambient exposure to PM_2.5_ under outdoor atmospheric PM_2.5_ pollution (Jiang et al., 2020a; Su et al., 2020).

Our research team used the real-ambient exposure system to find that PM_2.5_ can cause multiple organ impairment, among which the lung damage was the most serious (Li et al., 2019a). Many studies have shown that PM_2.5_ contains complex toxic chemical components (Li et al., 2019a; Zhou et al., 2019). Pollutants in the air can be released into the lung surfactants and adhere to lung epithelial cells, causing lung injury. Previous studies have confirmed that PM_2.5_ exposure is highly associated with lung diseases such as pulmonary fibrosis, pneumonia, and chronic obstructive pulmonary disease, and the mechanism of lung damage by PM_2.5_ is mainly mediated by inflammation and oxidative stress (Guo et al., 2019).

Nrf2 is a vital transcription factor that regulates inflammation and antioxidant defense. Activation of the Nrf2/ARE signaling pathway can induce the expression of endogenous antioxidant enzymes and antioxidant proteins under normal conditions or under the stimulation of subtoxic doses (Ma, 2013). When the body is stimulated by PM_2.5_ exposure, Nrf2 can transfer from the cytoplasm to the nucleus and induce the transcription of antioxidant and anti-inflammation enzymes by binding to antioxidant response elements (AREs) (Suzuki and Yamamoto, 2015; Bellezza et al., 2018). Consequently, Nrf2 can protect the body against excessive oxidative stress damage. Several studies have reported that Nrf2-deficiency increases the effect of oxidative stress after exposure to PM_2.5_, causing cytotoxicity and inflammation in mice (Chen et al., 2018; Ge et al., 2020). However, another study confirmed that Nrf2 deficiency alleviates high-fat diet-induced liver damage by reducing PPARγ expression (Li et al., 2020b). Recent studies have also shown that Nrf2-knockout did not aggravate the harm caused by PM_2.5_ (Jiang et al., 2020b; Cui et al., 2020).

In this study, inflammatory and oxidative stress was increased in WT mice after exposure to PM_2.5_ and the lung function of WT-PM groups was decreased compared to WT-FA groups, and these results are consistent with other studies (Yue et al., 2019). However, there was no significant change in lung function or pathology. The level of inflammatory and oxidative stress was up-regulated between the KO-FA and KO-PM groups following PM exposure; this result may be related to the different roles played by Nrf2 in different situations. It is well-known that Nrf2 is a double-edged sword (Tebay et al., 2015; Wu et al., 2019a). In general, Nrf2 has a positive effect on body health. However, the target genes of Nrf2 might contribute to cell damage by promoting the metabolism of exogenous compounds, which suggests that the cytoprotective effect changes to promoting damage in certain contexts (Tebay et al., 2015). After short-term or sub-toxic dose stimulation, Nrf2 can regulate the expression of phase-Ⅱ metabolic enzymes and protect cells from oxidative damage. However, the expression of phase-Ⅱ metabolic enzymes is not sustained under long-term stimulation, which changes the cytoprotective effect of Nrf2 (Wu et al., 2019a).

To explore the lung damage effect of PM_2.5_ exposure in Nrf2-knockout mice, the RNA of all genes expressed in lung tissue was sequenced. RNA sequencing is characterized by the high-throughput screening for DEGs and it can uncover many DEGs after exposure to various toxicants (Shukla et al., 2017). In wild-type mice, PM_2.5_ exposure resulted in 4,908 DEGs in lung tissue relative to the FA control, which suggests that PM_2.5_ exposure induces a series of effects. As mentioned in the results, we studied 3,671 genes to determine the influence of Nrf2 loss and the top-10 KEGG signaling pathways were selected by RNA sequencing. One major mechanism of PM_2.5_ cardiotoxicity is mediated via oxidative stress. Previous studies have demonstrated that PM_2.5_ exposure has toxic effects on the heart such as cardiomyopathy and coronary disease (Pope et al., 2016; Qi et al., 2020). Nrf2 deletion was found to be related to cardiac injury but there was no connection between the pathways affected in cardiac damage and pulmonary injury. Among the various signaling pathways, the CYP450 signaling pathway was found to be closely related to lung injury in a previous study (Stading et al., 2021), and so the genes in the signaling pathway were selected and explored further. CYP450 participates in the metabolism of exogenous substances, including drugs and environmental compounds. It could metabolize PAHs attached to PM_2.5_, which can produce more toxic oxidation metabolites, leading to lung damage (Oesch et al., 2019). The mRNA expression levels of CYP2E1 in WT mice showed an increasing trend after PM_2.5_ exposure. However, the levels of CYP2E1 were down-regulated in the KO-PM group relative to the KO-FA group, suggesting that fewer toxic metabolites appeared in KO mice following exposure to PM_2.5_ and thus caused less damage to lung tissue.

CYP2E1 is a membrane protein expressed at a high level that is primarily distributed in the ER and mitochondrial membrane (Bièche et al., 2007; Hartman et al., 2017). It is closely related to ER stress, which may be why lung injury in KO mice is not obvious after PM exposure. Increasing amounts of evidence have indicated that CYP2E1-dependent ER stress contributes substantially to the pathogenesis of radiation-induced pulmonary fibrosis (Son et al., 2017) and environmental toxicant-induced liver toxicity (Wu et al., 2019b). In addition, the ER is a key organelle that causes dysfunction and affects the response of other cell structures such as the mitochondria, cytoplasm, and the nucleus (Lin et al., 2019). If ER stress continues, it will lead to apoptosis and necrotic cell death (Wu et al., 2019b). Many studies have shown that ER stress is involved in body damage caused by PM_2.5_ (Zhou et al., 2017; Familari et al., 2019). As markers protein of ER stress and the most abundant ER glycoprotein, GRP94 and CHOP play pivotal roles in the maintenance of protein homeostasis and participate in the ER unfolded protein response (Ghiasi et al., 2019; Klymenko et al., 2019).

Our results indicate an increased expression level of GRP94 and CHOP in WT mice after exposure to PM_2.5_, suggesting that ER stress induced by PM_2.5_ metabolites may take part in the pathogenesis of lung damage. However, the decreased expression of GRP94 and CHOP in PM-treated Nrf2-knockout mice suggests that the decreased expression of CYP2E1 in KO mice means that they have a weakened metabolic activation capacity of exogenous compounds, which reduces their production of toxic metabolites. As a result, the ER stress was decreased in KO-PM group. According to previous study, persistent inflammation and oxidative stress caused by PM_2.5_ exposure can lead to lung damage in mice (Cantin et al., 2015). In our study, the level of inflammation and oxidative stress were up-regulated in WT mice after exposure to PM_2.5_. And the ER stress was also increased in WT-PM mice compared with WT-FA mice. Previous study has shown that ER stress is involved in ventilator-induced lung injury in mice via the IRE1α-TRAF2-NF-κB pathway (Ye et al., 2020). Therefore, the lung function and pathology showed significant changes in WT mice after exposure to PM_2.5_. In the KO mice, the level of inflammation and oxidative stress were increased under PM_2.5_ exposure. However, the ER stress was decreased significantly in KO-PM mice compared with KO-FA mice. According to our study, the decrease of ER stress in KO-PM mice may be due to the down-regulated of CYP2E1 expression by Nrf2. This may explain the lack of changes in lung function and pathology in the KO-FA group.

**FIGURE 6:**
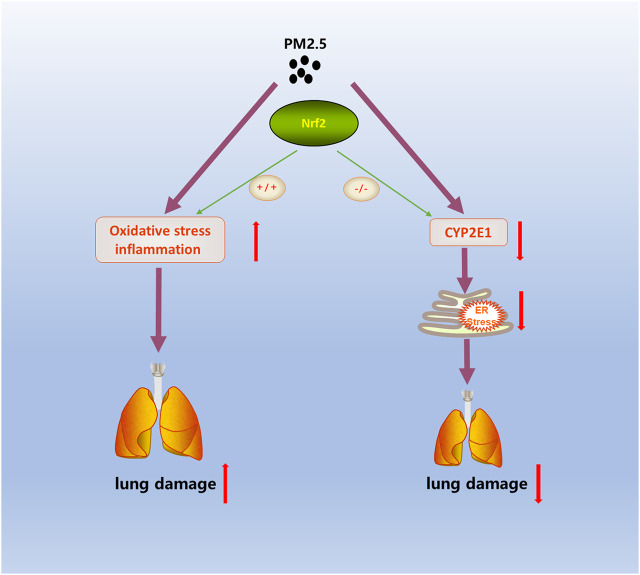
Scheme of the Nrf2-mediated lung injury induced by PM_2.5_.

In the present study, we established a novel exposure model named the “real-ambient exposure” system. This inhalational model completely simulates the whole-body inhalation of outdoor PM_2.5_. Our study indicated an adverse effect on lung function under PM_2.5_ exposure and revealed the underlying mechanism of CYP2E1 metabolism in Nrf2-deficient mice (Figure 6). Our results further broaden our recognition of the harmful impact of PM_2.5_ and provide a new mechanism for the process.

## Data Availability

The raw data supporting the conclusion of this article will be made available by the authors, without undue reservation, to any qualified researcher.
