# Supplementary material for: Effects of Real-Ambient PM2.5 Exposure on Lung Damage Modulated by Nrf2−/−
Source: Front Pharmacol. 2021 Apr 23;12:662664. doi: 10.3389/fphar.2021.662664 (PMC8104929; doi:10.3389/fphar.2021.662664)
Supplement: Supplementary file 1 [file DataSheet1.docx]

***Supplemental material***

**Contents**

**Table S1.** Primer pairs used in *q*RT-PCR.

**Figure S1.** The expression levels of Nrf2 was evaluated by western blot.

**Figure S2.** The mRNA expression level of HO-1, NQO1 and GCLC.

**TableS1.** Primer sequences used in *q*RT-PCR.

| Species | Gene | Primer sequence |  |
| --- | --- | --- | --- |
| Mouse | CYP2e1 | Forward | AGGCTGTCAAGGAGGTGCTACT |
|  |  | Reverse | AAAACCTCCGCACGTCCTTCCA |
| Mouse | β-actin | Forward | CATTGCTGACAGGATGCAGAAGG |
|  |  | Reverse | TGCTGGAAGGTGGACAGTGAGG |
| Mouse | CYP2s1 | Forward | TGTCGTTGACGCCTTCCTGCTA |
|  |  | Reverse | GCAAACAGCAGGTATGTGACCG |
| Mouse | Gsta4 | Forward | CGGCCAAGTACCCTTGGTTGAAAT |
|  |  | Reverse | AATGGAGCCACGGCAATCATCATC |
| Mouse | Mgst1 | Forward | TGCGACCGCATTCCAGAGGATA |
| Mouse  Mouse  Mouse  Mouse  Mouse | GRP94  CHOP  HO-1  NQO1  GCLC | Reverse  Forward  Reverse  Forward  Reverse  Forward  Reverse  Forward  Reverse  Forward  Reverse | TCCACCTTCTCGTCAGTGCGAA  AAGGTCATTGTCACGTCGAAA  GTGTTTCCTCTTGGGTCAGC  GGAGGTCCTGTCCTCAGATGAA  GCTCCTCTGTCAGCCAAGCTAG  CACTCTGGAGATGACACCTGAG  GTGTTCCTCTGTCAGCATCACC  GCCGAACACAAGAAGCTGGAAG  GGCAAATCCTGCTACGAGCACT  ACACCTGGATGATGCCAACGAG  CCTCCATTGGTCGGAACTCTAC |


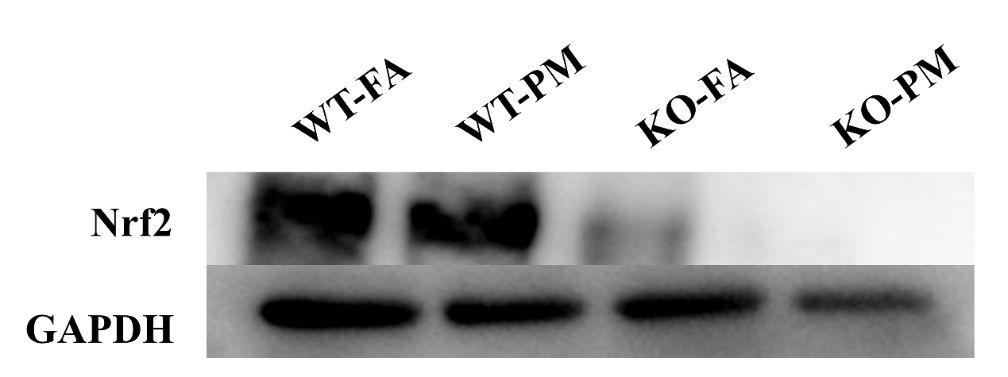

**FigS1.** The expression levels of Nrf2 was evaluated by western blot and quantified with ImageJ (NIH, United States) software.


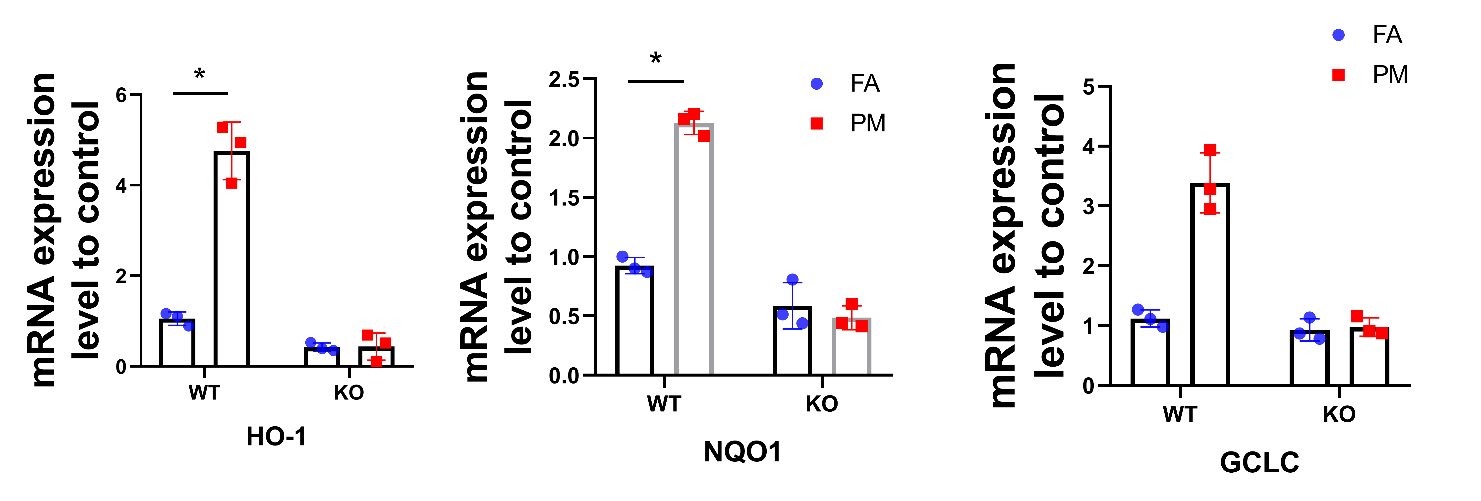


**FigS2.** The mRNA expression level of HO-1, NQO1 and GCLC. N=3 per group. FA, filtered air; PM, fine particulate matter. WT, wild type mice; KO, Nrf2^-/-^ mice. Data are expressed as the mean ± SEM, *p< 0.05.
